# Immune Regulation of *Plasmodium* Is *Anopheles* Species Specific and Infection Intensity Dependent

**DOI:** 10.1128/mBio.01631-17

**Published:** 2017-10-17

**Authors:** Maria L. Simões, Godfree Mlambo, Abhai Tripathi, Yuemei Dong, George Dimopoulos

**Affiliations:** W. Harry Feinstone Department of Molecular Microbiology and Immunology, Malaria Research Institute, Bloomberg School of Public Health, Johns Hopkins University, Baltimore, Maryland, USA; EPFL

**Keywords:** *Anopheles*, *Plasmodium*, innate immunity, melanization, malaria

## Abstract

Malaria parasite ookinetes must traverse the vector mosquito midgut epithelium to transform into sporozoite-producing oocysts. The *Anopheles* innate immune system is a key regulator of this process, thereby determining vector competence and disease transmission. The role of *Anopheles* innate immunity factors as agonists or antagonists of malaria parasite infection has been previously determined using specific single *Anopheles*-*Plasmodium* species combinations. Here we show that the two C-type lectins CTL4 and CTLMA2 exert differential agonistic and antagonistic regulation of parasite killing in African and South American *Anopheles* species. The C-type lectins regulate both parasite melanization and lysis through independent mechanisms, and their implication in parasite melanization is dependent on infection intensity rather than mosquito-parasite species combination. We show that the leucine-rich repeat protein LRIM1 acts as an antagonist on the development of *Plasmodium* ookinetes and as a regulator of oocyst size and sporozoite production in the South American mosquito *Anopheles albimanus*. Our findings explain the rare observation of human *Plasmodium falciparum* melanization and define a key factor mediating the poor vector competence of *Anopheles albimanus* for *Plasmodium berghei* and *Plasmodium falciparum*.

## IMPORTANCE

Malaria, one of the world’s deadliest diseases, is caused by *Plasmodium* parasites that are vectored to humans by the bite of *Anopheles* mosquitoes. The mosquito’s innate immune system is actively engaged in suppressing *Plasmodium* infection. Studies on mosquito immunity revealed multiple factors that act as either facilitators or inhibitors of *Plasmodium* infection, but these findings were mostly based on single *Anopheles*-*Plasmodium* species combinations, not taking into account the diversity of mosquito and parasite species. We show that the functions of CTL4 and CTLMA2 have diverged in different vector species and can be both agonistic and antagonistic for *Plasmodium* infection. Their protection against parasite melanization in *Anopheles gambiae* is dependent on infection intensity, rather than the mosquito-parasite combination. Importantly, we describe for the first time how LRIM1 plays an essential role in *Plasmodium* infection of *Anopheles albimanus*, suggesting it is a key regulator of the poor vector competence of this species.

## INTRODUCTION

Transmission of the malaria protozoan parasite *Plasmodium* to the vertebrate host involves a complex infection cycle in the mosquito vector that includes sexual sporogonic development through several stages. As it feeds upon blood, the female mosquito ingests *Plasmodium* gametocytes that mature into gametes within the midgut lumen; fertilization of these gametes produces zygotes, which in turn develop into motile ookinetes. Ookinetes invade and traverse the mosquito midgut epithelium at about 18 to 36 h post-blood meal (PBM). After reaching the basal side of the epithelium, they develop into oocysts, maturation of which involves several mitotic divisions that result in thousands of sporozoites. Sporozoites are released into the hemocoel at about 14 days PBM, and from here they invade and infect the salivary glands. Sporozoites are then transmitted into the vertebrate host during a subsequent blood meal, thus beginning the asexual portion of the *Plasmodium* infection cycle.

Vector competence, a measure of susceptibility to *Plasmodium*, can vary greatly among different strains and populations of *Anopheles gambiae sensu stricto* (s.s.), as well as among different members of the species complex. The genetic variability responsible for these differences has in some cases been linked to the mosquito’s innate immune system and shown to be dependent on specific immune gene alleles (1–3). Furthermore, a certain *A. gambiae sensu lato* species, or even strain, can differ in its vector competence for different *Plasmodium* species or isolates (4–6). For example, the genetically selected *A. gambiae* L3-5 strain (7) is refractory to the rodent parasite *Plasmodium berghei* through melanotic encapsulation of the ookinetes in the midgut epithelium; on the other hand, this strain can support infection with the African human parasite *Plasmodium falciparum*. We have previously shown that the *A. gambiae* molecular responses to infection with either *P. berghei* or *P. falciparum* parasites differ greatly at the level of gene transcript abundance, as well as with regard to immune gene function (8, 9).

Insects lack an adaptive immune system, and thus they rely solely on innate immunity for their defense against various microorganisms, including *Plasmodium*. Melanization is a prime immune reaction of insects, for which the *A. gambiae* L3-5 line mentioned above has become a laboratory model. However, it is a very rare phenomenon in susceptible *A. gambiae* strains; melanization in these strains is almost never observed in the laboratory and only rarely seen in the field (10–16).

A landmark in the study of mosquito immunity to *Plasmodium* infection was the discovery of two *P. berghei* agonists, the *A. gambiae* C-type lectins CTL4 (AgCTL4) and CTLMA2 (AgCTLMA2), which were shown to promote the development of the rodent malaria parasite *P. berghei* in the African mosquito vector *A. gambiae* by protecting the malarial ookinetes from melanization (12). In the same study, an *A. gambiae* leucine-rich repeat protein, AgLRIM1, was found to be a *P. berghei* antagonist, exerting a potent antiparasitic effect that involved lysis, but not melanization, of the parasite (12). Most laboratory studies on immunity to *Plasmodium* infection, including those investigating the involvement of AgCTL4, AgCTLMA2, and AgLRIM1, have relied on specific *A. gambiae* and *Plasmodium* strains and species but have not extensively addressed possible differences attributable to genetic variation in either organism. In addition, the majority of the literature references to the function of these three genes describe their role in the context of mosquito infection with the rodent parasite *P. berghei*.

The findings of AgCTL4, AgCTLMA2, and AgLRIM1 activity in regulating anti-*Plasmodium* immunity (12) were performed with the *A. gambiae* G3 laboratory strain, which originated from a mixture of populations of M, S, or hybrid M/S molecular forms of *A. gambiae* (17) and is considered susceptible to *P. berghei* (18). This discovery was only followed up by one study with *P. falciparum* (15) that utilized the field-collected *A. gambiae* Yaoundé strain, membrane fed with human donor blood infected with natural field isolates of *P. falciparum*. This study did not detect any effect of silencing any of the three genes on infection with the human malaria parasite. Together, the studies by Osta et al. (12) and Cohuet et al. (15) suggested that AgCTL4 and AgCTLMA2 play an agonistic role in and influence the melanization of *P. berghei*, but not *P. falciparum*, and that AgLRIM1 is an antagonist of *P. berghei* alone. The suggestion was made that the differences between infection phenotypes observed upon *P. berghei* and *P. falciparum* infection of C-type lectin- and LRIM1-silenced *A. gambiae* mosquitoes reflect the coevolution of the human parasite *P. falciparum* and the immune system of its natural vector, *A. gambiae* (15, 19–21). Indeed, *P. falciparum* shares a geographic and evolutionary history with *A. gambiae*, contrarily to *P. berghei* (22). However, a potentially important difference between the two studies, apart from the parasite species and mosquito strains utilized, is the fact that *P. berghei* achieves unnaturally high infection intensities in *A. gambiae*, frequently exceeding 200 oocysts per mosquito, but the experiments by Cohuet et al. (15) showed median infection intensities for *P. falciparum* of only 2 to 3 oocysts per mosquito. Another study (23) has suggested that *Anopheles* responses to *Plasmodium* are dependent on the intensity of infection.

The vast majority of studies on mosquito immunity to *Plasmodium* have focused on the *A. gambiae* infection system, and little is known about anti-*Plasmodium* immunity of *Anopheles albimanus*, one of the most important New World human malaria vectors. This species belongs to the subgenus *Nyssorhynchus*, which is thought to have diverged from subgenus *Cellia* (malaria vectors from Africa, India, and South Asia) about 100 million years ago, when the African and South American continents separated (24, 25). *A. albimanus* is likely one of the first native mosquito vectors that African *P. falciparum* encountered upon its arrival in the New World (26). Previous studies have shown that *A. albimanus* is distinctly more refractory to *P. berghei* and *P. falciparum* oocyst infection than are other mosquito species. Indeed, *P. berghei* had initially been reported to be incapable of infecting *A. albimanus* (27) or to infect this vector at a low prevalence and intensity (28). Likewise, the results of experiments involving the *P. falciparum* strain NF54 have suggested that *A. albimanus* is a poor vector (29, 30), and in the 3D7 strain, ookinetes completely failed to mature and develop into oocysts (31).

In the present study, we have explored the roles of the key mosquito innate immunity factors CTL4, CTLMA2, and LRIM1 in the vector competence of various *Anopheles* species and strains for various *Plasmodium* species, revealing novel conditions and features of the anti-*Plasmodium* defense, melanization, and evolution of mosquito immunity. We show for the first time that the melanization response in *CTL4*- and *CTLMA2-*silenced *A. gambiae* mosquitoes is dependent on infection intensity, rather than parasite species. Moreover, we also reveal that *CTL4* and *CTLMA2* have diverged in terms of their function in *A. albimanus*, where they play *Plasmodium*-antagonistic roles. We further show that LRIM1 is a regulator of oocyst and sporozoite development in *A. albimanus*, thereby explaining the low vector competence of this species for *Plasmodium*, as described by several earlier studies.

## RESULTS

### The melanization response in *CTL4*- and *CTLMA2-*silenced *A. gambiae* is dependent on infection intensity.

**FIG 1:**
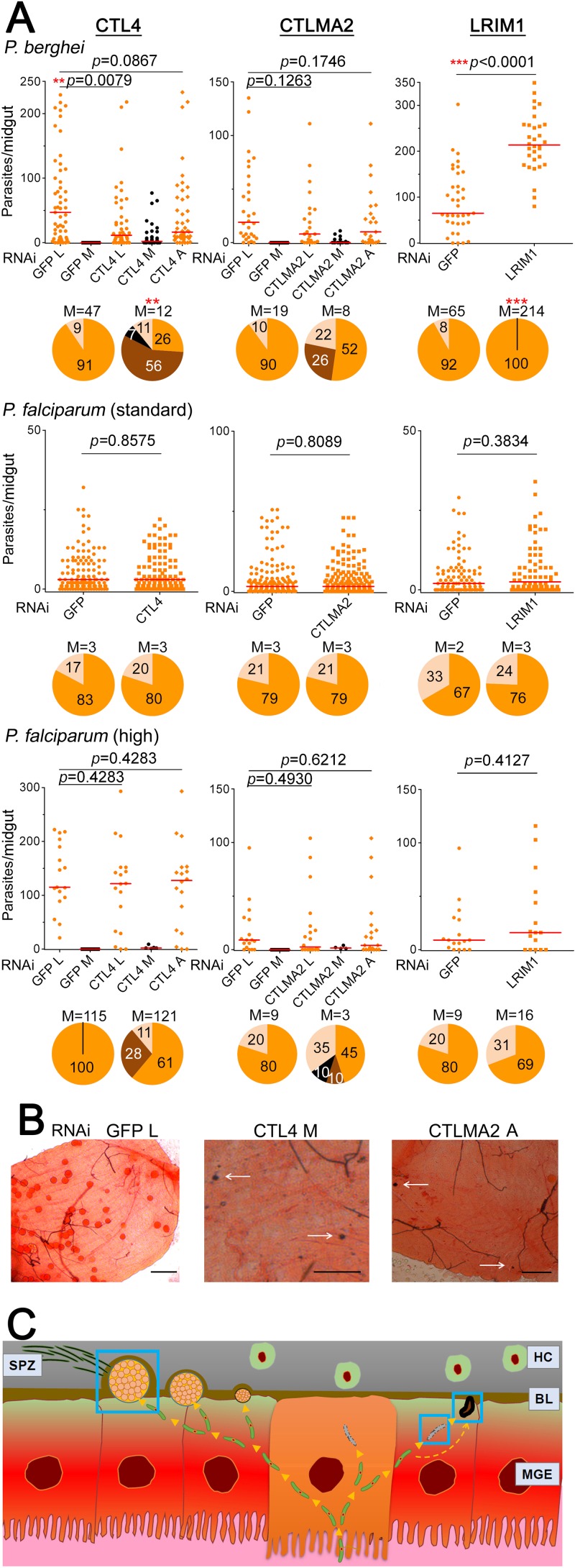
*Plasmodium* infection in *A. gambiae*. (A) Dots indicate the number of parasites in the individual midguts of female *A. gambiae* (Keele strain) mosquitoes infected with *P. berghei* (standard infection) or *P. falciparum* (standard and high infection). L, live parasites (orange dots); M, melanized parasites (black dots); A, all parasites (sum of L and M parasites). Horizontal red bars indicate the median. Pie charts show the percentage of *A. gambiae* midguts not infected (clear), containing live parasites only (orange), melanized parasites only (black), or containing both live and melanized (dark brown) parasites. Median (M) numbers of oocysts are shown above the pie charts. Two-tailed *P* values by Mann-Whitney test are shown: **, *P* < 0.01; ***, *P* < 0.001. Detailed statistical information concerning the infection assays is summarized in Table S2. (B) Images illustrate *P. falciparum*-infected *A. gambiae* midguts showing live parasites, melanized parasites (indicated by white arrows), and both live and melanized parasites. Scale bars, 100 µm. (C) A model of the parasite phenotypes observed in this study during the *Plasmodium* invasion of the *A. gambiae* midgut. Blue squares indicate the phenotypes observed. From left to right are shown a regular-size *A. gambiae* oocyst, a lysed ookinete, and a melanized ookinete. MGE, midgut epithelium; BL, basal lamina; HC, hemocytes; SPZ, sporozoites.

To address the possible mosquito and parasite strain-specific dependency of the roles of AgCTL4, AgCTLMA2, and AgLRIM1 in anti-*Plasmodium* defense, we first investigated the effects of gene silencing on *P. berghei* infection in the *A. gambiae* Keele strain. This strain was established by interbreeding four laboratory colonies: the West African G3 strain and another three strains originating from East Africa (32). RNA interference (RNAi)-mediated silencing of *AgCTL4* and *AgCTLMA2* resulted in a reduced intensity of infection with *P. berghei* (as measured by live oocyst numbers), and melanization was observed in 63% of *AgCTL4*-silenced and 26% of *AgCTLMA2-*silenced mosquitoes (Fig. 1A). Moreover, in 7% of the *AgCTL4*-silenced *A. gambiae* Keele midguts, every single parasite was melanized (Fig. 1A), suggesting that gene function with regard to *P. berghei* infection is not mosquito strain specific. The median *P. berghei* infection intensity of nonsilenced mosquitoes (green fluorescent protein double-stranded RNA [dsRNA]-treated [dsGFP] control) varied from 19 to 65, and the number of oocysts per mosquito ranged from 0 to 302 (Fig. 1A). As seen in the study using the *A. gambiae* G3 strain (12), *AgLRIM1*-silenced Keele strain mosquitoes showed a significantly increased (*P* < 0.0001) infection intensity compared to the controls, with a median of 214 oocysts per midgut (Fig. 1A), confirming this gene’s conserved antagonistic action against *P. berghei* infection among *A. gambiae* strains. We and others have suggested that LRIM1 is required for melanization in *A. gambiae* (5, 20, 33), and as expected, its inactivation resulted in a complete lack of melanization of *P. berghei* parasites (Fig. 1A).

After confirming the conserved functions and influences of the three genes on *P. berghei* infection in the *A. gambiae* Keele strain, we next addressed their influence on infection with the human malaria parasite *P. falciparum*. We infected two *A. gambiae* laboratory lines, Keele and G3, with the *P. falciparum* NF54 strain following RNAi-mediated silencing of the three genes. Compared to the *P. berghei* infections, the *P. falciparum* parasite loads were much lower, with a median number of oocysts per group (including the GFP dsRNA-treated control), of 2 to 3 oocysts per midgut for the Keele strain (Fig. 1A) and 0 to 7 for the G3 strain mosquitoes (see Fig. S1 in the supplemental material). This low intensity of infection mimics the natural infection conditions observed in the study by Cohuet et al. (15), and the results of our assays were also similar to those of that study. Indeed, no significant differences in *P. falciparum* infection intensity or prevalence were observed between the control and gene-silenced mosquitoes of the two mosquito strains (Fig. 1A; Fig. S1). Furthermore, no melanized ookinetes were detected in any of the mosquito cohorts.

10.1128/mBio.01631-17.1 FIG S1  *Plasmodium* infection in the *A. gambiae*, G3 strain. Dots indicate the number of parasites in individual midguts of female *A. gambiae* (G3 strain) infected with *P. falciparum* (standard and high infection). L, live parasites (orange dots); M, melanized parasites (black dots); A, all parasites (sum of L and M parasites). Horizontal red bars indicate the median. Two-tailed *P* values by Mann-Whitney test are shown. Pie charts show the percentage of *A. gambiae* (G3 strain) midguts not infected (clear), containing live parasites only (orange), or containing both live and melanized parasites (dark brown). The median (M) number of oocysts is shown above the pie charts. Detailed statistical information concerning the infection assays is summarized in Table S2. Download FIG S1, TIF file, 1.5 MB. Copyright © 2017 Simões et al. 2017 Simões et al. This content is distributed under the terms of the Creative Commons Attribution 4.0 International license.

Next, we hypothesized that perhaps the observed differences between the *P. berghei* and *P. falciparum* infection phenotypes of gene-silenced mosquitoes were dependent on infection intensity rather than parasite species. To address this possibility, we infected gene-silenced *A. gambiae* Keele mosquitoes using a higher *P. falciparum* gametocytemia that yielded median infection intensities ranging from 9 to 115 in the GFP dsRNA-treated control group and oocyst numbers per mosquito ranging from 0 to 222 (Fig. 1A and B). A previous study had also shown a correlation between gametocytemia and oocyst density (23). Under these conditions, we observed, for the first time, *P. falciparum*-melanized parasites (Fig. 1A and C) in the *AgCTL4* gene-silenced mosquitoes, with a melanization prevalence of 28% (Fig. 1A and B), and melanization was also observed when *AgCTLMA2* was silenced (Fig. 1A and B). The same phenotype was observed when we used a high level of *P. falciparum* infection in *AgCTL4*-silenced *A. gambiae* G3 mosquitoes (15% of midguts showed melanized ookinetes) (Fig. S1). Our results agree with the correlation previously observed between responses to *P. berghei* and a high intensity of *P. falciparum* infection (23). Feeding mosquitoes on high numbers of gametocytes did not result in melanized parasites in the *AgLRIM1-*silenced mosquitoes (Fig. 1A). Importantly, the results presented here show for the first time that the melanization of *P. falciparum* NF54 parasites in susceptible *A. gambiae* strains is infection intensity dependent and only consistently occurs at artificially generated high infection intensities, explaining why it has been so rarely observed in previous studies.

### The influence of *CTL4* and *CTLMA2* on *Plasmodium* infection has diverged between *A. albimanus* and *A. gambiae.*

After investigating the roles of *AgCTL4*, *AgCTLMA2*, and *AgLRIM1* in immune responses of the African malaria vector *A. gambiae* to human and rodent malaria parasites, we wanted to investigate whether the influences of these genes on *Plasmodium* infection were similar in the Central and South American vector *A. albimanus*. Such a similarity would indicate conservation of *Plasmodium* agonistic and antagonistic mechanisms across *Anopheles* species. We first identified the putative orthologs of *A. gambiae CTL4*, *CTLMA2*, and *LRIM1* in *A. albimanus* as *AALB014534*, *AALB005905*, and *AALB005865*, here referred to as *AaCTL4*, *AaCTLMA2*, and *AaLRIM1*, respectively.

**FIG 2:**
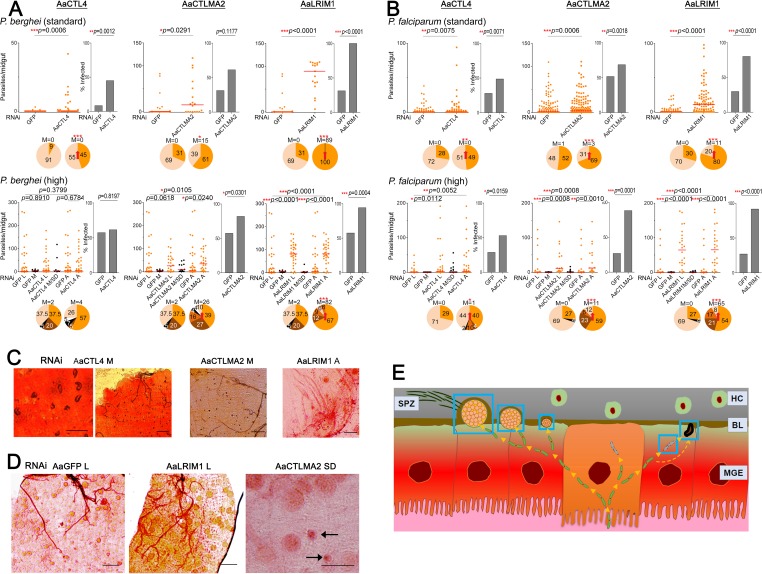
*Plasmodium* infection in *A. albimanus*. (A and B) Dots indicate the number of parasites in individual midguts of female *A. albimanus* infected with (A) *P. berghei* or (B) *P. falciparum*. L, live parasites; M, melanized parasites; M/SD, melanized and smaller and darker parasites; A, all parasites. Horizontal red bars indicate the median. Bars in the infection prevalence graphs show the percentage of mosquitoes harboring at least one oocyst. Pie charts show the percentage of *A. albimanus* midguts not infected (clear); containing live parasites only (orange), melanized parasites only (black), live and melanized parasites (dark brown), live, melanized, and smaller and darker parasites, live and smaller and darker parasites (light brown), or melanized and smaller and darker parasites (white). The median (M) number of oocysts is shown above each pie chart; red arrows indicate significant increase in prevalence (Fisher’s exact test). Two-tailed *P* values by Mann-Whitney test (infection intensity) or Fisher exact test (infection prevalence) are shown: *, *P* < 0.05; **, *P* < 0.01; ***, *P* < 0.001. (C) Images illustrate *P. berghei*-infected *A. albimanus* midguts showing melanized (M) and both live and melanized (A [all]) parasites. Scale bars are 20 µm in the far left image and 100 µm in the other images. (D) *P. falciparum*-infected *A. albimanus* midguts showing particular *A. albimanus* infection phenotypes: differently sized live parasites (left image), uniform and large live parasites (central image), or smaller and darker (indicated by black arrows) parasites (right image). Scale bars, 100 µm. (E) Model of the parasite phenotypes during the *Plasmodium* invasion of the *A. albimanus* midgut. Blue squares indicate the phenotypes observed. From left to right are shown a large *AaLRIM1*-silenced oocyst, a regular-size *A. albimanus* oocyst, a smaller and darker early oocyst, a lysed ookinete, and a melanized ookinete.

In agreement with previous studies, we observed that both *P. berghei* and *P. falciparum* had low basal levels of infection in the GFP dsRNA-treated control *A. albimanus* compared to *A. gambiae*. The median infection intensities of *A. albimanus* controls for both rodent and human malaria parasites rarely surpassed 0 oocyst per midgut (Fig. 2A and B), whereas in *A. gambiae* Keele, they ranged from 2 to 3 oocysts per midgut for *P. falciparum* and from 19 to 65 for *P. berghei* (Fig. 1A). However, for both parasite species, silencing of *AaCTL4*, *AaCTLMA2*, and *AaLRIM1* resulted in significantly different infection intensities, and in most cases also prevalences, from the controls (Fig. 2A and B), in sharp contrast to our results with *P. falciparum* infection in *A. gambiae* (Fig. 1A). Interestingly, our results also revealed that the two C-type lectins exhibited opposite functions between the Old World and New World vectors, since their silencing resulted in increased *P. berghei* and *P. falciparum* infection levels in *A. albimanus*. Specifically, the number of oocysts significantly increased when *AaCTL4* (*P* = 0.0006, *P* = 0.0075) and *AaCTLMA2* (*P* = 0.0291, *P* = 0.0006) were silenced and mosquitoes were infected with the murine or the human malaria parasite, respectively (Fig. 2A and B). Silencing of *AaLRIM1* also resulted in significantly increased infection (*P* < 0.0001) of both parasite species (Fig. 2A and B). Hence, in *A. albimanus*, the function o*f CTL4* and *CTLMA2* seems to have diverged from that in *A. gambiae*, whereas the antagonistic function of *LRIM1* seems to be conserved between the African and the American *Anopheles* species. Of note, silencing the three *A. albimanus* genes did not result in any parasite melanization in either *P. berghei* or *P. falciparum* in these standard-infection experiments.

We also explored the effect of high parasite infection intensity in *A. albimanus*. As shown in Fig. 2B, increasing the percentage of *P. falciparum* gametocytes also resulted in melanized ookinetes in the *AaCTL4*- and *AaCTLMA2*-silenced groups, as well as a low percentage of melanized ookinetes in the dsGFP control. Osta et al. (12) also observed *P. berghei* melanization in the control group in *A. gambiae* G3 strain experiments, but here we only observed this response in the control group in *A. albimanus* with high infection intensity. We also performed high-infection-intensity experiments with *P. berghei* by extending the amount of time the *A. albimanus* females were allowed to feed on parasite-infected mice. Melanized *P. berghei* parasites were also observed in highly infected *A. albimanus* mosquitoes. In fact, 17% of the mosquitoes showed melanized *P. berghei* ookinetes in the *AaCTL4*-silenced group, 51% in the *AaCTLMA2*-silenced group, and 25% in the control group (Fig. 2A and C). Interestingly, melanized ookinetes were also seen when *AaLRIM1*-silenced *A. albimanus* mosquitoes were infected with high-gametocytemic *P. berghei*- or *P. falciparum*-infected blood (21% or 38% of midguts with melanized ookinetes, respectively) (Fig. 2A to C). This infection phenotype was never observed when *LRIM1* was silenced in *A. gambiae*.

### *AaLRIM1* influences *Plasmodium* oocyst development and size and sporozoite development.

The Central-South American *A. albimanus* species has been considered a less efficient vector for *Plasmodium* than is the African *A. gambiae* (27–31). Grieco et al. (30) have also pointed out that the *P. falciparum* NF54 oocysts are smaller in *A. albimanus* than in *A. gambiae*, suggesting that there may be physiological factors that inhibit the oocysts’ development in *A. albimanus*. In the present study, the numerous parallel infection assays in the two mosquito species allowed for a direct comparison of oocyst and sporozoite infection phenotypes. Both the *P. berghei* and *P. falciparum* NF54 oocysts in nonsilenced and GFP dsRNA-injected control *A. albimanus*, as well as in the *AaCTL4*- and *AaCTLMA2*-silenced groups, were in general smaller than in similarly treated *A. gambiae* (Fig. 2D and E). The sizes of the oocysts in the high-intensity-infected GFP dsRNA-treated control as well as the *AaCTL4*- and *AaCTLMA2*-silenced *A. albimanus* were also irregular: differently sized oocysts were observed in the same midgut (Fig. 2D) more often than in *A. gambiae*.

Remarkably, in the *AaLRIM1*-silenced *A. albimanus*, the sizes of the oocysts were uniform and the oocysts were much larger than in the other *A. albimanus* groups (including the non-gene-silenced control) (Fig. 2D and E); the size was comparable to that of the oocysts in *A. gambiae* in both *Plasmodium* species. This observation suggests that *AaLRIM1* influences *Plasmodium* oocyst development in *A. albimanus* and that the smaller oocyst size in this mosquito species that was observed by us and others is, at least partially, a result of the action of *AaLRIM1*. Moreover, we have also observed parasites that were smaller and darker (SD) than the normal oocysts, but not yet melanized, in the *AaCTL4*-, *AaCTLMA2*-, and *AaLRIM1*-silenced and highly infected mosquitoes (Fig. 2A and B and D and E). We also examined *P. berghei* sporozoite phenotypes in *A. albimanus*. Previous studies have shown that the natural sporozoite infection rates in *A. albimanus* are usually extremely low (29, 30). At 21 days PBM, we observed sporozoites in 91% of the *P. berghei*-infected dsGFP control *A. gambiae* salivary glands, while only 4% of the dsGFP control *A. albimanus* mosquitoes showed sporozoites in their salivary glands (see Fig. S2 in the supplemental material). This pattern is in agreement with the study by Grieco and coworkers (30), which found no sporozoites in the salivary glands of one *A. albimanus* strain, whereas sporozoites were present in the salivary glands of 2.2% of mosquitoes of another strain. Contrarily, we observed that 60% of the salivary glands from the *AaLRIM1-*depleted mosquitoes were infected with sporozoites (Fig. S2). These data are in agreement with the midgut infection phenotypes of the gene-silenced *A. albimanus* that we described above. The increase in salivary gland sporozoite infection upon *AaLRIM1* silencing further indicates a key role for *AaLRIM1* in regulating oocyst development in *A. albimanus*, since its depletion rescued the low level of oocyst and sporozoite infection normally observed in this New World vector.

10.1128/mBio.01631-17.2 FIG S2  *P. berghei* sporozoite infection. Bars represent the percentage of salivary glands infected with sporozoites. Two-tailed *P* values by Fisher’s exact test: *, *P* < 0.05; ***, *P* < 0.001. Download FIG S2, TIF file, 0.6 MB. Copyright © 2017 Simões et al. 2017 Simões et al. This content is distributed under the terms of the Creative Commons Attribution 4.0 International license.

### *A. albimanus* lacks a true *A. gambiae* CTL4 ortholog.

**FIG 3:**
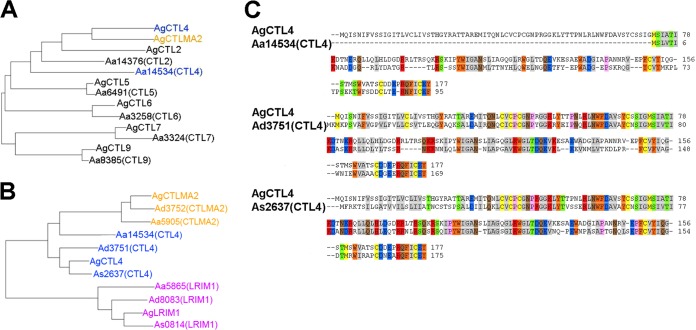
Evolutionary relationships in various mosquito species. (A and B) Phylogenetic trees (neighbor-joining) of (A) *A. gambiae* CTL family members and their putative *A. albimanus* orthologues and *A. gambiae* CTLMA2 and (B) *A. gambiae* CTL4, CTLMA2, and LRIM1 and their putative orthologs in *A. albimanus*, *A. dirus*, and *A. stephensi*. (Note that AgCTLMA2 has no ortholog in *A. stephensi*.) (C) Pairwise amino acid alignment of full-length *A. gambiae* CTL4 and its putative orthologs in *A. albimanus*, *A. dirus*, and *A. stephensi*.

In order to gain insight into the possible mechanistic basis for the differences in the impact of the C-type lectin silencing between the two vectors, we performed a phylogenetic analysis based on protein sequences retrieved from VectorBase and as defined by previous studies (34, 35). Alignment of *A. gambiae* and *A. albimanus* CTL family members together with AgCTLMA2, showed that AgCTL4 did not cluster in the same branch as its putative *A. albimanus* ortholog [Aa14534(CTL4)]. Instead, AgCTL4 clustered together with AgCTLMA2, whereas it is separated from its putative *A. albimanus* ortholog (Fig. 3A). This pattern was not observed upon alignment of CTLMA2 and LRIM1 family members of the two mosquito species; AgCTLMA2 and AgLRIM1 clustered in the same branch as their predicted orthologs in *A. albimanus* (see Fig. S3A in the supplemental material).

10.1128/mBio.01631-17.3 FIG S3  Evolutionary relationships in *A. gambiae* and *A. albimanus*. (A) Phylogenetic trees (neighbor-joining) of *A. gambiae* CTLMA subfamily members (left panel) and LRR long subfamily members (right panel) and their putative orthologs in *A. albimanus*. (B) Pairwise amino acid alignment of full-length *A. gambiae* LRIM1 and its putative ortholog in *A. albimanus*. Download FIG S3, TIF file, 0.7 MB. Copyright © 2017 Simões et al. 2017 Simões et al. This content is distributed under the terms of the Creative Commons Attribution 4.0 International license.

Next, we explored the phylogenetic relationships of CTL4, CTLMA2, and LRIM1 among the *Anopheles* species *A. gambiae* (African), *A. albimanus* (American), *Anopheles dirus* (Asian), and *Anopheles stephensi* (Asian). The three proteins clustered with their correspondent orthologs in the various species, except for CTL4’s predicted ortholog in *A. albimanus* [Aa14534(CTL4)], which branched separately from the AgCTL4, AdCTL4, and AsCTL4 cluster (Fig. 3B). This observation was supported by pairwise amino acid alignment of full-length AgCTL4 and its putative orthologs in *A. albimanus*, *A. dirus* (AdCTL4), and *A. stephensi* (AsCTL4), which showed a much lower degree of identity and similarity between the Ag/AaCTL4 amino acid residues (22% and 29%, respectively) compared to the other pairs (49% and 61% for Ag/AdCTL4, and 60% and 74% for Ag/AsCTL4) (Fig. 3C). These results show that AALB014534 (*AaCTL4*) is most likely not a true ortholog of *AgCTL4*, and they may also explain the differences in infection phenotypes of gene-silenced *A. gambiae* and *A. albimanus* after *Plasmodium* infection.

Rottschaefer and coworkers (36) have shown that CTL4 and CTLMA2 are located directly adjacent to each other on the *A. gambiae* 2L chromosome, and Schnitger et al. (37) have suggested that *A. gambiae* CTL4 and CTLMA2 may have evolved from a common ancestral gene by duplication and diversification, eventually leading to the formation of a disulfide-linked heterodimeric gene product that is functionally crucial. This hypothesis is also reflected by the clustering of AgCTL4 in the same branch as AgCTLMA2, whereas it is separated from its putative *A. albimanus* ortholog (Fig. 3A). These authors’ observations (36, 37) could explain the similar *Plasmodium*-agonistic roles of AgCTL4 and AgCTLMA2 that have been observed by us and by others (12), as well as the differences in infection phenotype upon gene silencing between *A. gambiae* and *A. albimanus*, given that the divergence of AaCTL4 may in some way abolish or change the CTLMA2-CTL4 heterodimer. Hence, our phylogenetic and functional data may corroborate the AgCTL4-AgCTLMA2 heterodimer hypothesis.

## DISCUSSION

Here we provide novel insights on how *Anopheles* malaria vectors regulate infection with malaria parasites through three key mosquito innate immunity factors, the C-type lectins CTL4 and CTLMA2 and the leucine-rich repeat protein LRIM1.

We found that silencing any of the three genes in the vector-parasite combination involving sympatric species, the *A. gambiae-P. falciparum* NF54 strain combination, did not affect the infection intensity or prevalence, corroborating previous results with other *A. gambiae* and *P. falciparum* strains (15). Contrarily, in the other vector-parasite pairs (*A. gambiae-P. berghei*, *A. albimanus-P. falciparum* NF54, and *A. albimanus-P. berghei*), depletion of the immunity genes resulted in significantly different infection phenotypes compared to the control. Hence, the recently proposed lock-and-key theory (38), a model suggesting that only the parasites expressing a *Pfs47* haplotype compatible (i.e., from the same geographical region) with a certain vector mosquito can evade anti-*Plasmodium* immunity, appears to partially explain immune evasion, which is also dependent on infection intensity, as proposed in our study and discussed below.

The study by Osta et al. (12) showing that depletion of *CTL4* and *CTLMA2* in the *A. gambiae* G3 strain results in melanotic encapsulation of *P. berghei* PbCTRPp.GFP ookinetes has established that gene system as a parasite melanization model. We also observed the same melanization phenotype in the *A. gambiae* Keele *CTL4*- and *CTLMA2*-silenced mosquitoes infected with the *P. berghei* ANKA 2.34 strain, which also achieves a high infection intensity; our findings corroborate previous observations using other mosquito and parasite strains (12, 39, 40) and thus confirm the conserved functions of these genes in various *A. gambiae* strains with regard to *P. berghei* infection. While these results are in agreement with the coevolution theory (*A. gambiae* and *P. berghei* did not evolve in geographical cohabitation), the melanization phenotype was not observed in *A. albimanus* when a standard infection intensity of the noncoevolved *P. berghei* was used, but only at a high infection intensity. Furthermore, our studies showed that *P. falciparum* melanization would always occur upon *CTL4* and *CTLMA2* silencing in *A. gambiae* and *A. albimanus* at high parasite infection intensities, as a result of the ingestion of a larger number of gametocytes.

Our findings thus shift the paradigm of parasite melanization dependence on noncoevolved mosquito-parasite species combinations to dependence on infection intensity; melanization is directly dependent on infection intensity and is not mosquito-parasite species dependent. It is possible that factors that are required for melanization only reach a critical threshold upon high infection intensity. The infection intensity-dependent infection phenotypes seen after C-type lectin silencing may be explained by the fact that RNAi-mediated gene silencing achieved only a certain degree of protein depletion, which in the case of a high-intensity infection would be sufficient to result in melanization because the remaining protein would not be sufficient to protect all the parasites. Conversely, when infection intensity is low, even the protein remaining after gene silencing would be sufficient to mask the much lower number of parasites from a melanization-mediated defense. Another possible contribution to the intensity-dependent melanization phenotype is the passage of bacteria through the injured epithelium upon high infection intensity. Studies have shown that the *A. gambiae* melanization response can be triggered by bacteria (41).

The opposite effects of *CTL4* and *CTLMA2* silencing on *P. berghei* infection intensity and prevalence that we observed between *A. gambiae* and *A. albimanus* strongly suggest that the functions of these proteins, or associated cofactors, have diverged. Importantly, these observations also indicate that mechanistic involvement of the two proteins in regulating infection intensity and melanization is unlinked. RNAi-mediated depletion of *AaCTL4* and *AaCTLMA2* also resulted in increased *P. falciparum* infection intensity in *A. albimanus*, while silencing of their *A. gambiae* orthologs did not alter infection intensity with the same parasite species, further supporting the functional divergence of these genes. It is possible that other components of the complex immune response network, which have differentiated between the two mosquito species, may influence implication of *AaCTL4* in immunity. However, the low sequence identity between *AgCTL4* and *AaCTL4*, taken together with the different gene-silencing phenotypes, strongly suggests that the two genes do not represent true orthologs. The two proteins likely function as a heterodimer in *A. gambiae* that may not be formed in *A. albimanus*. In fact, AaCTL4 does not contain the N-terminal cysteine residues involved in disulfide linkages between AgCTL4 and AgCTLMA2 (37).

Several studies have described the New World *A. albimanus* as an inefficient vector for *P. falciparum* and *P. berghei* because of its low infection intensity and small oocysts that do not produce sporozoites. *AgLRIM1* was initially described as an anti-*Plasmodium* effector that participates in ookinete-stage lysis. Here we show that *AaLRIM1* also compromises ookinete development (as indicated by increased oocyst numbers upon gene silencing) in *A. albimanus*, as well as oocyst size and sporozoite production, thereby differentiating its function from that of the *A. gambiae* ortholog. Whether these two *AaLRIM1*-silencing infection phenotypes are dependent on the same, or different, *AaLRIM1* functions is unclear. In addition, unlike the situation in *A. gambiae*, *AaLRIM1* does not seem to be required for melanization in the New World vector. The differences encountered in LRIM1 among the two mosquito species are supported by the low degree of identity and similarity of their amino acid residues (35% and 53%, respectively [Fig. S3B]).

In summary, our study of the key *Anopheles* immune factors CTL4, CTLMA2, and LRIM1 has revealed the following major novel facets of vector competence for the malaria parasites:

1. *A. gambiae* C-type lectin protection against parasite melanization is dependent on infection intensity, rather than the mosquito-parasite species combination.
2. *A. albimanus* LRIM1 is a key regulator of *Plasmodium* infection intensity, oocyst size, and sporozoite development, thereby explaining the poor vector competence of this vector for *P. berghei* and *P. falciparum*.
3. CTL4 and CTLMA2 have diverged in their functions between *A. albimanus* and *A. gambiae*, playing parasite antagonist and agonist roles in the two vector species, respectively.

## MATERIALS AND METHODS

### Ethics statement.

All animal work was conducted in strict accordance with the recommendations in the *Guide for the Care and Use of Laboratory Animals* of the National Institutes of Health. The protocols and procedures used in this study were approved by the Animal Care and Use Committee of the Johns Hopkins University (permit no. M006H300) and the Johns Hopkins School of Public Health Ethics Committee. Commercial anonymous human blood was used for parasite cultures and mosquito feeding; thus, informed consent was not required.

### RNAi-mediated gene silencing.

VectorBase (http://www.vectorbase.org) was consulted to identify the putative orthologs of *A. gambiae CTL4*, *CTLMA2*, and *LRIM1* in *A. albimanus*: *AALB014534*, *AALB005905*, and *AALB005865*. PCR products were generated from cDNA using gene-specific primers for each mosquito species that included a T7 promoter sequence. Each PCR product was purified and sequenced, and after sequence confirmation, specific dsRNA was synthesized using the HiScribe T7 Quick HighYield RNA synthesis kit (New England Biolabs) according to the manufacturer’s instructions. dsGFP was used as control dsRNA. dsRNA concentrations and quality were assessed by spectrometry and agarose gels. The sequences of primers used for dsRNA synthesis can be found in Table S1 in the supplemental material.

10.1128/mBio.01631-17.5 TABLE S1  Primers. The primers used for RNAi-mediated gene silencing are shown in the upper row, and those for real-time qRT-PCR analysis are shown in the lower row. dsRNA primers include the T7 promoter sequence. Download TABLE S1, DOCX file, 0.1 MB. Copyright © 2017 Simões et al. 2017 Simões et al. This content is distributed under the terms of the Creative Commons Attribution 4.0 International license.

### Mosquito rearing and injection.

*A. gambiae* s.s. and *A. albimanus* mosquitoes were reared and maintained under laboratory conditions as in reference 9. Adult mosquitoes were routinely fed on anesthetized 6- to 8-week-old female Swiss Webster mice for egg production. Three-day-old female mosquitoes were randomly taken from a population cage and were cold anesthetized and inoculated intrathoracically with 69 nl of a 3-μg/μl solution of dsRNA for each gene of interest. A control reference group was injected with dsGFP. All injections were repeated two to four times using a Nanoject microinjector, and at least 80 mosquitoes were silenced per group and per experiment. Each biological replicate corresponded to a different mosquito population cage, and each population corresponded to a different generation. After dsRNA injection, mosquitoes were left to rest for 3 to 4 days.

### Real-time qRT-PCR analysis.

Efficiency of gene silencing was assessed 3 to 4 days after dsRNA injection by real-time quantitative reverse transcription-PCR (qRT-PCR) for all genes tested and compared to the dsGFP-injected control mosquitoes. Total RNA was isolated (RNeasy kit; Qiagen), and cDNA was synthesized using 1 μg of total RNA with oligo(dT) primers and Moloney murine leukemia virus (MMLV) reverse transcriptase (Promega). Three replicates were used per gene and per mosquito species/strain. Quantitative analysis was performed by qRT-PCR using SYBR green PCR master mix (Applied Biosystems) in a final volume of 20 μl with a StepOnePlus real-time PCR system (Applied Biosystems). For all assays, the expression levels of target genes were normalized to the levels of ribosomal protein S7 gene (*AGAP010592* for *A. gambiae* or *AALB010399* for *A. albimanus*). The sequences of the primers used for silencing validation can be found in Table S1 (sequences for *AaS7* were retrieved from reference 38). Silencing efficiencies are shown in Fig. S4 in the supplemental material.

10.1128/mBio.01631-17.4 FIG S4  Efficiency of gene silencing. Silencing efficiency in dsRNA-injected mosquitoes was assessed by qRT-PCR. Bars represent the mean ± standard error (SE) percentage of gene expression following gene silencing in each mosquito species/strain compared to the dsGFP control. Download FIG S4, TIF file, 4 MB. Copyright © 2017 Simões et al. 2017 Simões et al. This content is distributed under the terms of the Creative Commons Attribution 4.0 International license.

### *Plasmodium* infection.

To determine the anti-*Plasmodium* activity, female mosquitoes were fed on an anesthetized *P. berghei* (ANKA 2.34 strain)-infected mouse or through artificial membrane feeders on a *P. falciparum* NF54 gametocyte culture in human blood. The *P. berghei* infectivity of each mouse was determined by measuring the parasitemia and observing at least one mature gametocyte and 1 to 2 exflagellations/field under the microscope. For high-infection *P. berghei* experiments, female mosquitoes were allowed to feed for a longer period on mice with higher parasitemia. Different *P. falciparum* NF54 gametocyte dilutions were used for high- and standard-infection exposures. After removal of the unfed females, *P. berghei*- and *P. falciparum*-infected *A. gambiae* and *A. albimanus* mosquitoes were kept for 8 to 10 days at 19°C (for *P. berghei*) and for 8 days at 27°C (for *P. falciparum*) for oocyst counting. Midguts were dissected in phosphate-buffered saline (PBS) and stained in 0.2% mercurochrome to determine oocyst numbers under a light-contrast microscope, and images were captured using an optical microscope. Sporozoites were counted at 21 days PBM in the salivary glands.

### Phylogenetic analysis.

The sequences of full-length *A. gambiae* CTL4 protein and its putative orthologs in *A. albimanus*, *A. dirus*, and *A. stephensi* were retrieved from VectorBase and aligned in the FASTA format, and amino acids were aligned pairwise (http://www.bioinformatics.org). Each amino acid residue was compared to the other residues in the same column, and identical or similar residues among the different species’ sequences were given a colored background according to their biochemical properties. The same procedure was used to verify the degree of identity and similarity between the *A. gambiae* LRIM1 protein and its putative *A. albimanus* ortholog. Phylogenetic analysis (neighbor-joining trees) was conducted in MEGA7 (42), using the protein sequences for CTL4, CTLMA2, and LRIM1 and their respective putative orthologs in the various mosquito species. For the *A. gambiae* CTL family members AgCTL1 to AgCTL9 (34), their sequences were aligned with those from the corresponding putative orthologs in *A. albimanus* (orthologs were found only for CTL2, -4, -5, -6, -7, and -9). Likewise, the members of the *A. gambiae* CTLMA subfamily and the members of the *A. gambiae* LRR long subfamily, to which AgLRIM1 belongs (35), were aligned with their putative orthologs in *A. albimanus*, and phylogenetic evolutionary analysis was conducted.

### Statistical analysis.

For each experimental treatment, the dot plots of the oocyst/ookinete numbers per midgut were generated using GraphPad Prism5 software. Bar graphs representing the percentage of mosquitoes harboring at least one oocyst were also generated by GraphPad Prism5 software. Pie charts were generated using Excel. Statistical differences between various biological replicates were tested, and when no differences were detected, similar numbers from different groups in each replicate were pooled. Significant differences in the infection intensity (the number of oocysts per individual midgut) and prevalence (the number of infected mosquitoes per total number of mosquitoes observed) between the dsGFP control and the gene-silenced groups were determined through the nonparametric Mann-Whitney test and the Fisher’s exact test, respectively, as in reference 43. Two-tailed *P* values are indicated for all experimental treatments. Information concerning the total number of midguts analyzed, median, range, prevalence, percentage of melanization, and *P* values for all treatments is presented in Table S2 in the supplemental material.

10.1128/mBio.01631-17.6 TABLE S2  Statistical analysis. The table shows detailed statistical information for all infection assays. Shown are the total number of midguts analyzed (N), median number of live oocysts, range of live oocysts, infection intensity and *P* value as measured by the Mann-Whitney test, infection prevalence (percentage) and *P* value as measured by Fisher’s exact test, and percentage of melanized midguts. Two-tailed *P* values: *, *P* < 0.05; **, *P* < 0.01; ***, *P* < 0.001. Download TABLE S2, DOCX file, 0.1 MB. Copyright © 2017 Simões et al. 2017 Simões et al. This content is distributed under the terms of the Creative Commons Attribution 4.0 International license.
