# Supplementary figures and images for: Immune Regulation of Plasmodium Is Anopheles Species Specific and Infection Intensity Dependent
Source: mBio. 2017 Oct 17;8(5):e01631-17. doi: 10.1128/mBio.01631-17 (PMC5646253; doi:10.1128/mBio.01631-17)

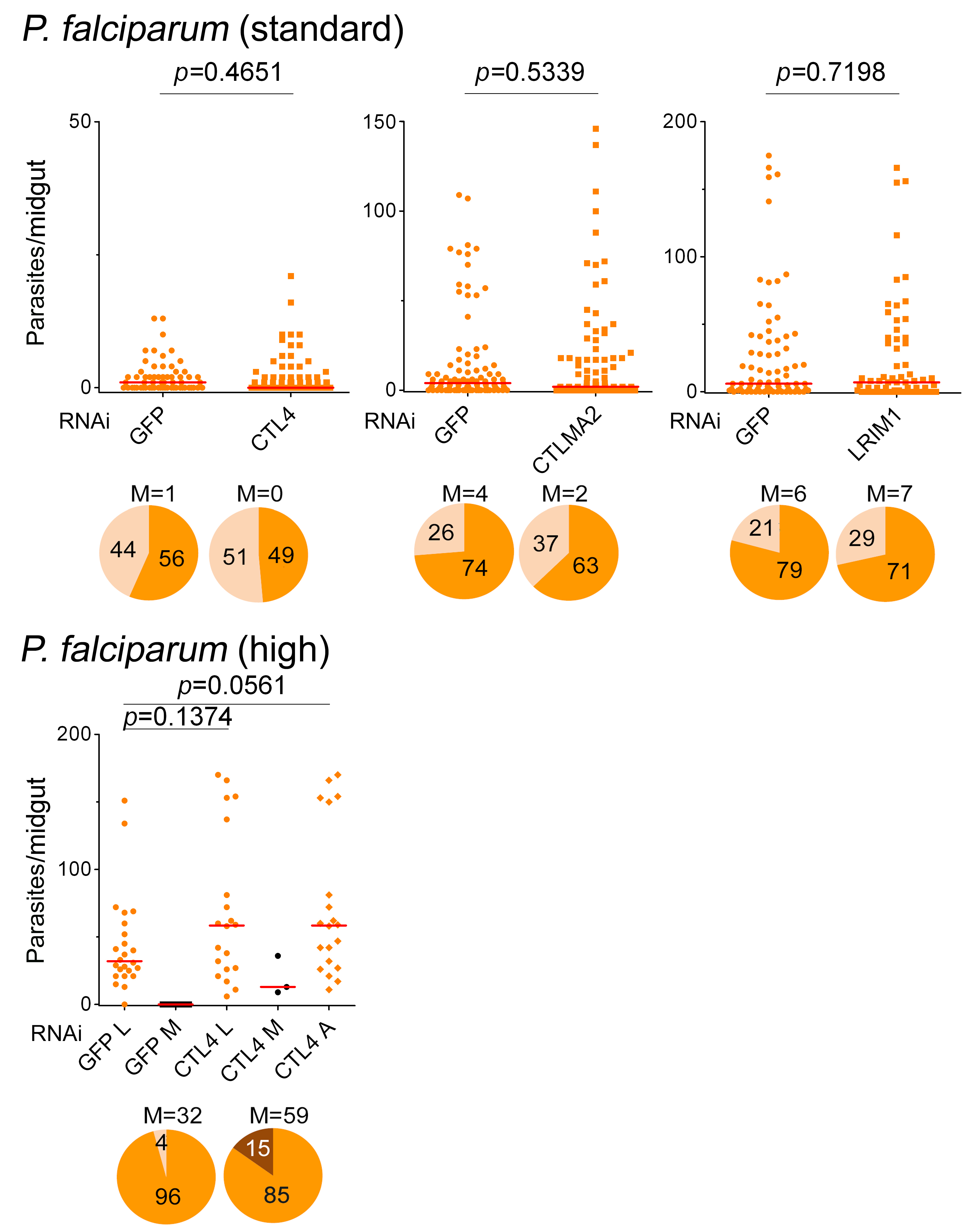

Supplement: FIG S1 [file mbo001173531sf1.tif]

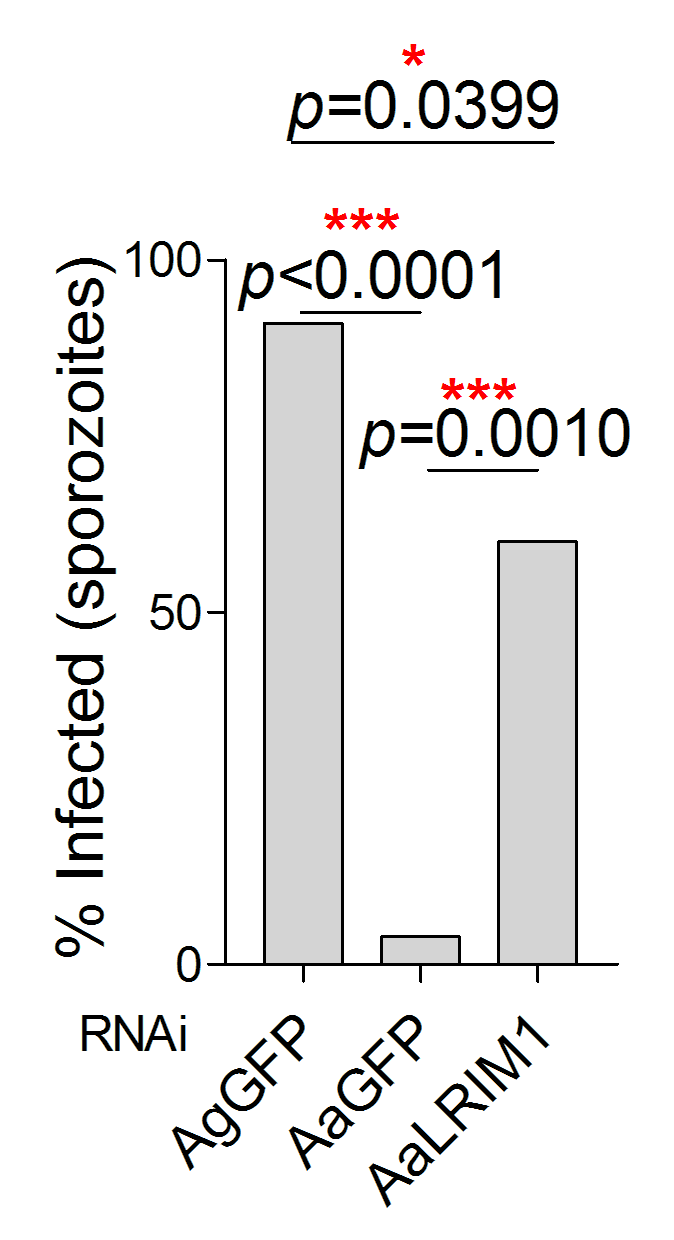

Supplement: FIG S2 [file mbo001173531sf2.tif]

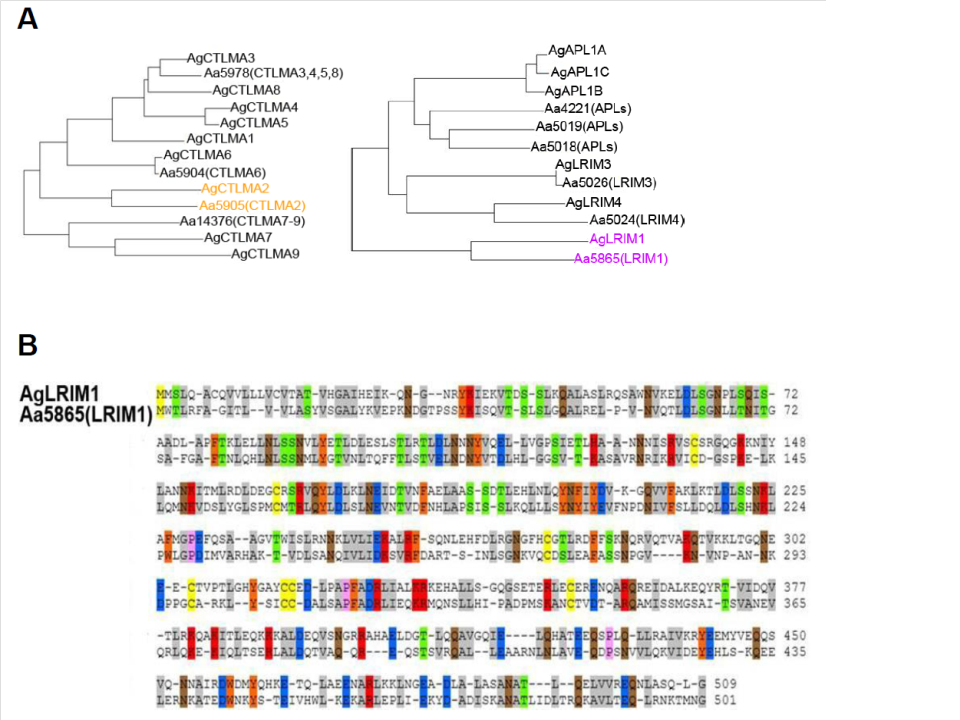

Supplement: FIG S3 [file mbo001173531sf3.tif]

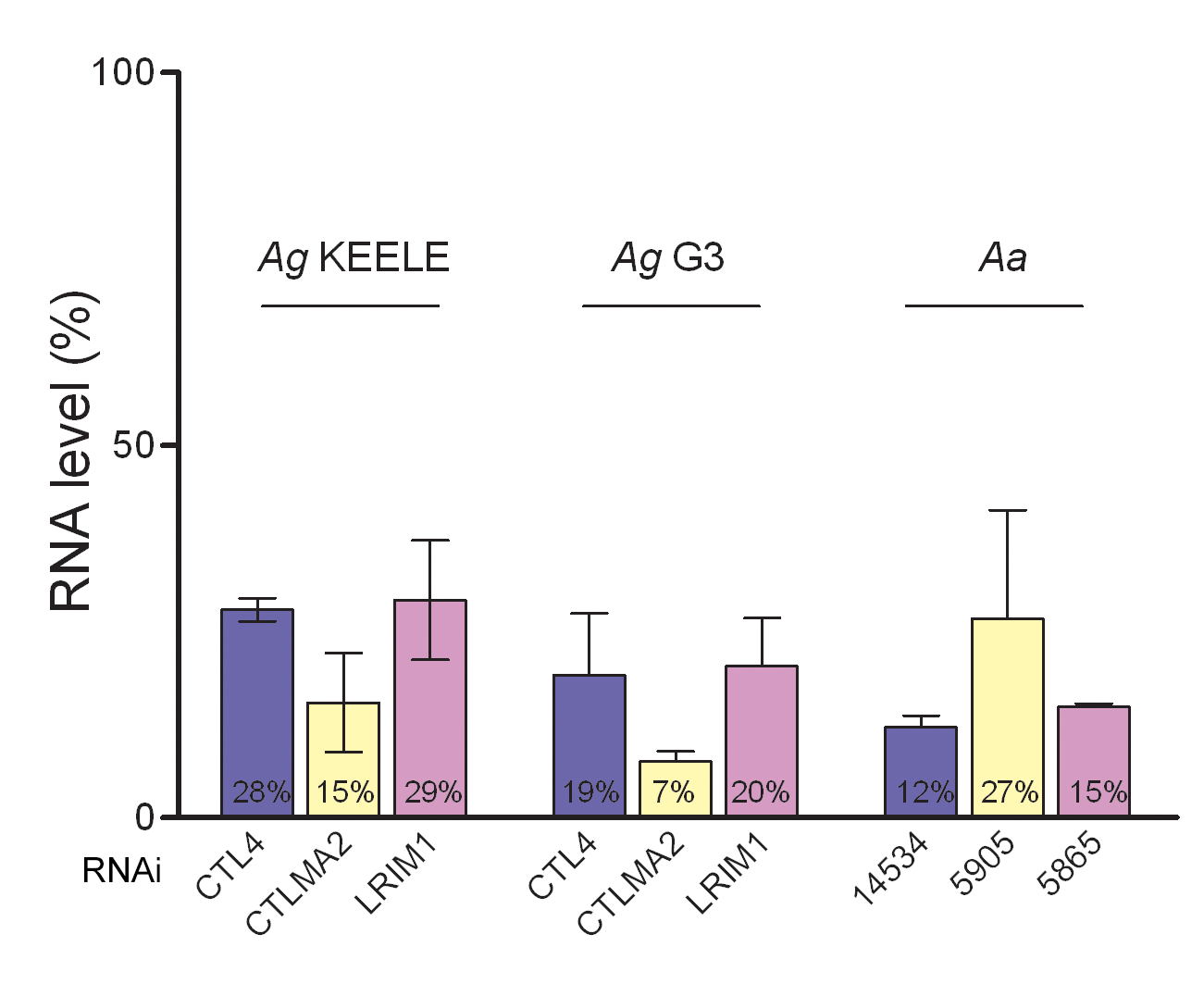

Supplement: FIG S4 [file mbo001173531sf4.tif]
