# Supplementary material for: Immune Regulation of Plasmodium Is Anopheles Species Specific and Infection Intensity Dependent
Source: mBio. 2017 Oct 17;8(5):e01631-17. doi: 10.1128/mBio.01631-17 (PMC5646253; doi:10.1128/mBio.01631-17)
Supplement: TABLE S1 [file mbo001173531st1.docx]

| **Table S1. Primers.** | | | |
| --- | --- | --- | --- |
| **Gene name** | **Accession number** | **Forward primer (5’-3’)** | **Reverse primer (5’-3’)** |
| *AgCTL4* | AGAP005335 | TAATACGACTCACTATAGGCAGCTCG | TAATACGACTCACTATAGGGAGTTTA |
|  |  | ATCGGAATGTCGATCGCTAC | TTCATCATCGCAAGAAGTCG |
| *AgCTLMA2* | AGAP005334 | TAATACGACTCACTATAGGGCAAACCGTTCGAGGAGAAAG | TAATACGACTCACTATAGGGTCTGCTGCGTACATTGGAAA |
|  |  | CACAGTGGTTCGTGGTGACCTA | CATGGGTTTTGTTGAAGAATATCATC |
| *AgLRIM1* | AGAP006348 | TAATACGACTCACTATAGGGCCAGAATGA | TAATACGACTCACTATAGGCAGCTCGATC |
|  |  | CGTGCCAAGTCGTCCTATTG | GGATTGCCGCTCAGATCAAG |
| *AaCTL4* | AALB014534 | TAATACGACTCACTATAGGTTCGTTCCTCGTCCTACTGG | TAATACGACTCACTATAGGGCTCCGTAAGGCAATCATCT |
|  |  | ACCTCTGCAGCGCGCAACTC | TCTGCCCGTCGCGTCGTAGA |
| *AaCTLMA2* | AALB005905 | TAATACGACTCACTATAGGGCAGCTGCGACGTAATGAAAA | TAATACGACTCACTATAGGGTCACCGATCTGGGCATTATT |
|  |  | GTGGCCGCTCAGTTCTATCT | TCACCGCTAATAAGCCCAAC |
| *AaLRIM1* | AALB005865 | TAATACGACTCACTATAGGGTTGCCGGCATTACTTTGGTC | TAATACGACTCACTATAGGGACCGTGCTGAGAGTGAAGAA |
|  |  | GACAAAAGTGTGCGCTTTGA | GAGGAGCGAGTTTTGCATTC |
| *GFP* |  | TAATACGACTCACTATAGGGTTCATCTGCACCACCGGC | TAATACGACTCACTATAGGGCTGGTAGTGGTCGGCGAG |
| *AgS7* | AGAP010592 | AGAACCAGCAGACCACCATC | GCTGCAAACTTCGGCTATTC |
| *AaS7* | AALB010399 | ACCTGGACAAGAACCAGCAG | GTTTTCTGGGAATTCGAACG |
