# Supplementary material for: Immune Regulation of Plasmodium Is Anopheles Species Specific and Infection Intensity Dependent
Source: mBio. 2017 Oct 17;8(5):e01631-17. doi: 10.1128/mBio.01631-17 (PMC5646253; doi:10.1128/mBio.01631-17)
Supplement: TABLE S2 [file mbo001173531st2.docx]

| **Table S2. Statistical analysis.** | | | | | | | | | | | | |
| --- | --- | --- | --- | --- | --- | --- | --- | --- | --- | --- | --- | --- |
| **Gene** | **Mosquito** | **Parasite** | **Infection** | **GFP Oocyst**  **Infection Intensity** | | **Gene Oocyst**  **Infection Intensity** | | **Intensity**  ***MW*** | **Prevalence**  **%** | | **Melanization**  **%** | |
|  |  |  |  | **N, Median** | **Range** | **N, Median** | **Range** |  | **GFP** | **Gene,**  ***Fisher*** | **GFP** | **Gene** |
| CTL4 | Ag K | Pb | Standard | 53, 47 | 0-229 | 46, 12 | 0-218 | 0.0079 (**), decrease | 91 | 82, 0.38 | 0 | 63 |
| CTL4 | Ag K | Pf NF54 | Standard | 122, 3 | 0-32 | 115, 3 | 0-22 | 0.86 | 83 | 80, 0.70 | 0 | 0 |
| CTL4 | Ag K | Pf NF54 | High | 17, 115 | 21-222 | 18,121 | 0-293 | 0.43 | 100 | 89, 0.49 | 0 | 28 |
| CTL4 | Ag G3 | Pf NF54 | Standard | 62, 1 | 0-13 | 72, 0 | 0-21 | 0.46 | 56 | 49, 0.46 | 0 | 0 |
| CTL4 | Ag G3 | Pf NF54 | High | 24, 32 | 0-151 | 20, 59 | 6-170 | 0.14 | 96 | 100, 0.93 | 0 | 15 |
| CTL4 | Aa | Pb | Standard | 35, 0 | 0-3 | 29, 0 | 0-42 | 0.0006 (***), increase | 9 | 45, 0.0012 (**), increase | 0 | 0 |
| CTL4 | Aa | Pb | High | 40, 2 | 0-260 | 39, 4 | 0-178 | 0.9 | 58 | 62, 0.82 | 25 | 17 |
| CTL4 | Aa | Pf NF54 | Standard | 118, 0 | 0-38 | 70, 0 | 0-94 | 0.0075 (**), increase | 28 | 49, 0.0071 (**), increase | 0 | 0 |
| CTL4 | Aa | Pf NF54 | High | 38, 0 | 0-49 | 50, 1 | 0-191 | 0.0112 (*), increase | 29 | 53, 0.0159 (*), increase | 0 | 16 |
|  |  |  |  |  |  |  |  |  |  |  |  |  |
| CTLMA2 | Ag K | Pb | Standard | 30, 19 | 0-135 | 23, 8 | 0-111 | 0.13 | 90 | 78, 0.43 | 0 | 26 |
| CTLMA2 | Ag K | Pf NF54 | Standard | 141, 3 | 0-51 | 158, 3 | 0-46 | 0.81 | 79 | 79, 0.95 | 0 | 0 |
| CTLMA2 | Ag K | Pf NF54 | High | 17, 9 | 0-95 | 20, 3 | 0-104 | 0.49 | 80 | 55, 0.31 | 0 | 20 |
| CTLMA2 | Ag G3 | Pf NF54 | Standard | 95, 4 | 0-109 | 91, 2 | 0-146 | 0.53 | 74 | 63, 0.14 | 0 | 0 |
| CTLMA2 | Aa | Pb | Standard | 24, 0 | 0-83 | 18, 15 | 0-117 | 0.0291 (*), increase | 31 | 61, 0.12 | 0 | 0 |
| CTLMA2 | Aa | Pb | High | 40, 2 | 0-260 | 26, 26 | 0-142 | 0.06 | 58 | 82, 0.0301(*),  increase | 25 | 51 |
| CTLMA2 | Aa | Pf NF54 | Standard | 178, 1 | 0-109 | 156, 3 | 0-110 | 0.0006 (***), increase | 52 | 69, 0.0018 (**), increase | 0 | 0 |
| CTLMA2 | Aa | Pf NF54 | High | 26, 0 | 0-123 | 17, 11 | 0-146 | 0.0008 (***), increase | 27 | 88, 0.0001  (***), increase | 4 | 29 |
|  |  |  |  |  |  |  |  |  |  |  |  |  |
| LRIM1 | Ag K | Pb | Standard | 37, 65 | 0-302 | 32, 214 | 80-349 | <0.0001 (***), increase | 92 | 100, 0.29 | 0 | 0 |
| LRIM1 | Ag K | Pf NF54 | Standard | 93, 2 | 0-29 | 78, 3 | 0-34 | 0.38 | 67 | 76, 0.91 | 0 | 0 |
| LRIM1 | Ag K | Pf NF54 | High | 17, 9 | 0-95 | 16,16 | 0-116 | 0.41 | 80 | 69, 0.91 | 0 | 0 |
| LRIM1 | Ag G3 | Pf NF54 | Standard | 77, 6 | 0-175 | 63, 7 | 0-166 | 0.72 | 79 | 71, 0.38 | 0 | 0 |
| LRIM1 | Aa | Pb | Standard | 24, 0 | 0-83 | 17, 89 | 6-109 | <0.0001 (***), increase | 31 | 100, <0.0001  (***), increase | 0 | 0 |
| LRIM1 | Aa | Pb | High | 40, 2 | 0-260 | 34, 82 | 0-169 | <0.0001 (***), increase | 58 | 94, 0.0004  (***), increase | 25 | 21 |
| LRIM1 | Aa | Pf NF54 | Standard | 104, 0 | 0-24 | 91, 11 | 0-98 | <0.0001 (***), increase | 30 | 80, <0.0001  (***), increase | 0 | 0 |
| LRIM1 | Aa | Pf NF54 | High | 26, 0 | 0-123 | 24, 65 | 0-177 | <0.0001 (***), increase | 27 | 92, <0.0001  (***), increase | 4 | 38 |
